# The Cause and Consequence Relationships between Domestic Abuse and Alzheimer's Disease: Identification of Five Subtypes

**DOI:** 10.1002/alz70858_099108

**Published:** 2025-12-25

**Authors:** Emma Twiss, Carley McPherson, Donald F Weaver

**Affiliations:** ^1^ Krembil Research Institute, Toronto, ON, Canada; ^2^ Lakeridge Health, Oshawa, ON, Canada; ^3^ University of Toronto, Toronto, ON, Canada

## Abstract

Domestic abuse (DA) and Alzheimer's Disease (AD; and related dementias) are two of humankind's most significant societal healthcare issues: DA is widespread, with one in three women and one in four men experiencing physical, psychological, emotional, sexual and/or financial abuse in their lifetime; AD is the most common form of dementia and will affect more than 152 million people worldwide by 2050. Given the incidence and prevalence of these two problems, any cause or effect relationship between them carries profound societal consequences. Herein, we describe five types of overlapping relationships between DA and AD: 1. Intimate partner violence as a risk factor for AD; 2. AD as a risk factor for ongoing or worsening DA; 3. Abuse of caregivers by people with AD; 4. Abuse of people with AD by caregivers; and 5. Reactivation of DA behavior in a person with AD. Chronologically, these five types cover the spectrum from occurring decades before the start of AD symptoms to onset only after the AD symptoms have manifested. Mechanistically, these five subtypes reflect the paradoxical fact that DA and AD may be the cause or consequence (or both) of each other. Phenomenologically, they encompass the full spectrum of DA including physical, psychological, emotional, sexual and financial abuse. Given the challenges in recognizing, managing and treating both DA and AD, society's need to identify and prevent these five subtypes of DA/AD overlap is an emerging priority.

